# Temporal and spatial changes in macrozoobenthos diversity in Poyang Lake Basin, China

**DOI:** 10.1002/ece3.5207

**Published:** 2019-04-26

**Authors:** Ke Li, Xiongjun Liu, Yu Zhou, Yang Xu, Qian Lv, Shan Ouyang, Xiaoping Wu

**Affiliations:** ^1^ School of Life Sciences Nanchang University Nanchang China; ^2^ Poyang Lake Key Laboratory of Environment and Resource Utilization Ministry of Education School of Resource Environment and Chemical Engineering Nanchang University Nanchang China; ^3^ School of Resource Environment and Chemical Engineering Nanchang University Nanchang China; ^4^ Center for Watershed Ecology Institute of Life Science Nanchang University Nanchang China

**Keywords:** assemblage structure, environmental elements change, macrozoobenthos, Poyang Lake

## Abstract

Poyang Lake plays a significant role in maintaining and replenishing the macrozoobenthos biodiversity in the middle Yangtze River. However, due to human activities and natural factors, the habitat of Poyang Lake has been seriously degraded, resulting in a decline in macrozoobenthos biodiversity. Here, we analyzed the effect of human activity and environmental elements change on the diversity of macrozoobenthos based on a systematic investigation of Poyang Lake Basin in 2016–2017. The current species richness, density, and biomass of macrozoobenthos were lower than those in the historical period. At the same time, the community structure of the macrozoobenthos assemblage exhibits significant temporal and spatial differences. In addition, the spatial turnover component was the main contribution to beta diversity, which indicated that a number of protected areas would be necessary to conserve the biodiversity of macrozoobenthos. Water depth, dissolved oxygen, water velocity, and chlorophyll‐a were significantly correlated with macrozoobenthos distributions and assemblage structure based on RDA. These results indicated that human activities have seriously destroyed the macrozoobenthos habitat and led to the decline in macrozoobenthos diversity. Therefore, habitat restoration and the conservation of macrozoobenthos have become urgent in Poyang Lake Basin, and an integrated management plan should be developed and effectively implemented.

## 1 INTRODUCTION

Habitat degradation is one of the most important factors for the decline of aquatic biodiversity (Dirzo & Raven, 2003; Kerr & Deguise, 2004; Krauss, Bommarco, & Guardiola, 2010; Wu, Huang, Han, Xie, & Gao, 2003). Lakes are important aquatic ecosystems and fishery habitats (Xie, 2017). There are unique shallow lakes with high habitat heterogeneity around the world, especially in the middle and lower reaches of the Yangtze River (Fu, Wu, Chen, Wu, & Lei, 2003; Xie, 2017). This region contains the most abundant fishery resources and freshwater species diversity in China, which plays an important role in maintaining the stability and security of the regional aquatic ecosystem and providing important ecosystem services for the development of human society and economy (Pan, Wang, Liang, & Wang, 2011; Wang & Dou, 1998). In recent decades, due to the effects of human activities and natural factors, such as climate change, dam construction, sand mining, and water pollution, lake area is not only shrinking and undergoing habitat fragmentation, but aquatic biodiversity is also being threatened (De Silva, Abery, & Nguyen, 2007; Vörösmarty et al., 2010; Zhang, Cai, & Qu, 2017).

Poyang Lake is the largest river‐connected lake in the Yangtze River (Xie, 2017), and plays a significant role in maintaining and replenishing aquatic biodiversity for the Yangtze River (Jin, Nie, Li, Chen, & Zhou, 2012). However, the impoundment of the Three Gorges Dam in 2003 changed the river–lake relationship between the Yangtze River and Poyang Lake, which affected the aquatic ecosystem of Poyang Lake (Min & Zhan, 2012; Zhang,Chen et al., 2015). At the same time, the habitat of Poyang Lake has been seriously degraded due to the effects of changes in anthropogenic habitats, resulting in the decline of aquatic biodiversity (Jin et al., 2012; Li, Zhang, Xia, & Gao, 2011; Xiong, Ouyang, & Wu, 2012; Zhang et al., 2013). Therefore, it is urgent to restore and protect the habitat and biodiversity of Poyang Lake.

Macrozoobenthos have proven to be an efficient indicator group in monitoring water quality and ecological integrity of ecosystems (Covich, Palmer, & Crowl, 1999; Saxena, 2014; Vanni, 2002), since they are sensitive to habitat changes, exhibiting weak migration, are easy to collect, and reflect long‐term changes in ecosystems (Chen, Bao, & Zhou, 2009; Zhang et al., 2011; Zhang, Lius et al., 2015). While several studies are limited in the area studied, their lack of systematic investigation, principal mechanisms relating diversity changes, and the way that anthropogenic habitats have changed interactions with range shifts in riverine systems have not been fully articulated in Poyang Lake (Cai et al., 2014; Ouyang, Zhan, Chen, Wu, & Wu, 2009; Wang, Xie, Wu, & Liang, 1999; Xie, Li, & Xiong, 1995). Here, we systematically investigated the macrozoobenthos community structure in Poyang Lake Basin during 2016 and 2017. Our specific aims were to (a) analyze the spatial and temporal changes in macrozoobenthos diversity and (b) determine how key environmental parameters and macrozoobenthos fauna varied in both spatial and temporal changes. We hope our study will provide an important reference for protecting the health and biodiversity of the ecosystem in Poyang Lake.

## 2 MATERIAL AND METHODS

### 2.1 Study area

**Figure 1:**
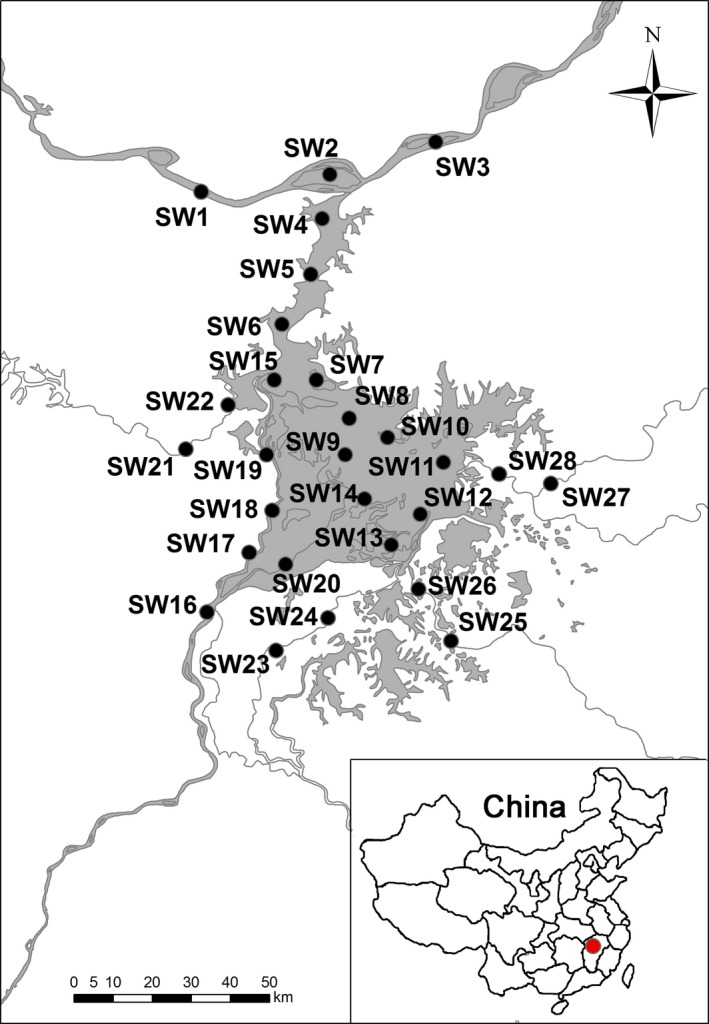
Location of the sampling sections in Poyang Lake Basin

Poyang Lake (28°22′–29°45′N, 115°47′–116°45′E), which is in northern Jiangxi Province and the southern bank of the middle Yangtze River, is the largest freshwater lake in China. It is surrounded on three sides by mountains, fed by five large rivers (Ganjiang River, Fuhe River, Xiuhe River, Xinjiang River, and Raohe River), and flows into the Yangtze River, forming a complex and highly interconnected river–lake–wetland system (Figure 1; Jin et al., 2012). The total area of Poyang Lake Basin is 16.2 × 10^4^ km^2^, which accounts for 9% of the Yangtze River Basin and 93.9% of the land area of Jiangxi Province. It has an average annual precipitation of 1,350–2,150 mm. Its surface runoff is 1,457 × 10^8^ m^3^, which accounts for 15% of the total runoff of the Yangtze River (Table 1). An annual average sediment load of 2,104.2 × 10^4^ ton flows into Poyang Lake, mainly from the five rivers. The average discharge into Poyang Lake is 4,690 m^3^, and the average discharge of exit is 4,700 m^3^, accounting for 16.8% of the annual average discharge (2,8300 m^3^/s) of Datong Station of the Yangtze River in 1956–2014 (Figure S1). In addition, the average water level of Poyang Lake ranges from 23.4 to 66.4 m in 2016–2017 (Figure S2). Poyang Lake is also a dynamic wetland system with a high water level in the rainy season of summer and a low water level in the dry season of winter. The difference in water depth is as high as 13 m in each year. During the rainy season from April to September, the floodplain is flooded, forming a large lake covering more than 3,000 km^2^. During the dry season from October to March, the submerged area of the lake can be reduced to <1,000 km^2^, forming a narrow zigzag passage (Cai et al., 2014).

**Table 1:** Temporal and spatial changes in species numbers of macrozoobenthos in Poyang Lake Basin

Study area	Code	Drainage area (km^2^)	Annual average temperature (°C)	Annual average precipitation (mm)	Length (km)	Human activity	Species number				
							Winter	Spring	Summer	Autumn	Total
Ganjiang River	GJ	82,809	18.3	1,580.8	766	Dam construction; water pollution; sand mining; overfishing shellfish; urban development	4	10	18	8	29
Xiuhe River	XH	14,797	16.7	1,663.2	419	Water pollution; sand mining	2	7	5	14	21
Fuhe River	FH	16,493	17.8	1,732.2	348	Water pollution; overfishing shellfish	6	3	33	17	32
Xinjiang River	XJ	17,599	17.8	1,855.2	359	Sand mining; overfishing shellfish	7	5	17	14	24
Raohe River	RH	15,300	17.3	1,849.7	299	Water pollution; sand mining; overfishing shellfish	3	5	12	5	18
Northern area of Poyang Lake	NL	4,125	17.2	1,541.8	—	Sand mining	4	13	15	12	26
Central area of Poyang Lake	CL					Sand mining; eutrophication	10	9	8	10	14
Southern area of Poyang Lake	SL					Sand mining; eutrophication; overfishing shellfish; drought	6	18	4	13	28
Connected‐river channel of Poyang Lake	TJ					Sand mining; drought; urban development	4	22	14	8	29
Yangtze River	YR	1,800,000	17.5	1,100	6,397	Industrial pollution; sand mining; urban development	4	6	2	4	12
Total							23	40	51	41	81

### 2.2 Sampling sites

In this study, sampling sites were selected by considering habitat variation and anthropogenic activities in Poyang Lake Basin. We established twenty‐eight sections (72 sampling sites) in Poyang Lake Basin (Figure 1), and each section was subdivided into three sampling sites that included (a) the middle reach of the Yangtze River: SW1‐SW3; (b) the connected‐river channel of Poyang Lake: SW4‐SW6; (c) the northern area of Poyang Lake: SW7‐SW8, SW15; (d) the central area of Poyang Lake: SW9‐SW11; (e) the southern area of Poyang Lake: SW12‐SW14; (f) the lower reach of the Ganjiang River: SW16‐SW20; (g) the lower reach of the Xiuhe River: SW21‐SW22; (h) the lower reach of the Fuhe River: SW23‐SW24; (i) the lower reach of the Xinjiang River: SW25‐SW26; (k) the lower reach of the Raohe River: SW27‐SW28.

### 2.3 Sampling methods

Three repeated samples of macrozoobenthos were collected using a modified Petersen grab (area of 1/16 m^2^) in the middle reach of the Yangtze River, the connected‐river channel of Poyang Lake, the lower of the “five rivers” and the main lake area of Poyang Lake in December (winter) 2016 and April (spring), July (summer), and October (autumn) 2017. In addition, three repeated samples of macrozoobenthos were obtained from the lower reach of “five rivers” using a Surber sampler (30 × 30 cm, 500 μm mesh). Sediment samples were sieved using a 500‐μm copper mesh sieve. The samples were maintained in a low temperature incubator and transported to the local laboratory. The specimens were carefully hand‐picked from the sediment on a white porcelain plate and later stored and preserved in 10% formaldehyde. Specimens were identified to the lowest possible taxonomic level (usually the species or genus level), counted, weighed, and converted to ash‐free dry weights with an electronic balance (HANGPING FA1204B; precision: 0.1 g) using relevant references (Yan & Liang, 1999; Zhao, Wang, Wang, & Liu, 2009). The macrozoobenthos taxonomic levels were mainly based on Liu, Zhang, and Wang (1993), Morse, Yang, and Tian (1994), Dudgeon (1999), Wang (2002a,2002b), and Tang (2006). The three replicate samples per site from a modified Petersen grab and a Surber sampler were pooled and then converted to density per square meter prior to the statistical analyses, respectively.

### 2.4 Measurement of physicochemical parameters

To analyze the changes in environmental factors in the study area, we measured physicochemical parameters in 28 sampling sections in December (winter) 2016 and April (spring), July (summer), and October (autumn) 2017. A YSI 650MDS (made in USA) multiparameter meter was used to measure the dissolved oxygen (DO), hydrogen ions (pH), turbidity (TURB), and water temperature (T). The chlorophyll‐a (Chl‐a) was measured with a chlorophyll meter (HL‐168C06, made in China), the water velocity was measured with a velocity meter (FP111, Global Water, 0.1 m/s accuracy), and the water depth was measured with a digital sonar system (H22px handheld sonar system).

### 2.5 Data analysis

The completeness of the macrozoobenthos species in each sampling section was assessed using abundance‐based rarefaction as implemented in iNext online (Chao, Ma, & Hsieh, 2016). Confidence intervals (95%) were calculated using 100 bootstrap replications.

The relative abundance of each species at each sampling site was estimated by:

(1) $$P_{i}=N_{i}/\sum_{\left(j=1\right)}^{s}N_{j}$$

where *S *= number of species, and *N*_*i*_ and *N*_*j*_ are the numbers of individual species in the sample. The Shannon–Wiener index (*H’*:* H’ *= −∑*P*_***i***_ln*P*_***i***_), Simpson index (*D*_*s*_: *D*_*s*_ *= *1‐∑(*P*_*i*_)^2^), and Pielou evenness index (*J’*:* J’ *=* H’*/ln*S*) were used to calculate macrozoobenthos species richness in each section (Magurran, 1988; Peet, 1974), where *S = t*he total number of species in each sample collected in the river.

Beta diversity represents the difference in species composition between different communities and is determined by species turnover and nestedness (Baselga, 2010; Carvalho, Cardoso, & Gomes, 2012). To quantify the effects of two processes, Baselga (2010) systematically proposed the beta diversity decomposition method (BAS frameworks) based on the Sørensen index (*β*_sor_), which was decomposed into species spatial turnover components (*β*_sim_) and nestedness components (*β*_sne_). Here, we analyzed the macrozoobenthos biodiversity based on the BAS frameworks.

BAS frameworks (Sørensen index):

$$\beta_{\mathrm{sor}}=\frac{b+c}{2a+b+c}$$

$$\beta_{\mathrm{sim}}=\frac{\min(b,c)}{a+\min(b,c)}$$

(2) $$\beta_{\mathrm{sne}}=\frac{\left|b-c\right|}{2a+b+c}\times \frac{a}{a+\min(b,c)}$$

where *a* is the number of shared species among two streams, and *b* and *c* are the number of species present in only the first and second streams, respectively. The Sørensen indices range from 0 to 1, representing situations in which no species and all species are common among two streams.

To explore the potential mechanisms of changes in beta diversity, we performed Mantel tests (Legendre & Legendre, 2012) with 9999 permutations to assess the correlations (Spearman's method) between pairwise dissimilarity matrices and the matrices of geographical distance. The geographical distances were measured among pairs of basins by measuring the distances between waterways in ArcMap GIS (ESRI). All beta diversity analyses were performed in R 3.2.0 (R Development Core Team, 2014) using the BETAPART package (Baselga & Orme, 2012) and VEGAN (Oksanen et al., 2015).

One‐way analysis of variance (ANOVA) was used to detect differences in water‐based and ecological environmental indices for macrozoobenthos (species number, density, biomass, Margalef index, Pielou's evenness index, Shannon‐Wiener index, and Simpson index) between each section and each season. The ANOVA tests were performed in SPSS 22.0.

Multidimensional scaling (MDS) was separately performed based on density to visualize changes in benthic assemblages (Clarke & Gorley, 2006). MDS was run in PRIMER 6 (Clarke & Gorley, 2006).

We used redundancy analysis (RDA) to evaluate variations in density in relation to environmental variables (ter Braak & Verdonschot, 1995; Lep & Smilauer, 2003). To show the importance of explaining the total variability in the density, we entered all variables into the analysis after a forward selection procedure. Monte Carlo permutation tests were used with 499 permutations to assess the significance (*P *<* *0.05) of the RDA gradient, and the eigenvalues of the first 2 axes were used to measure their importance (ter Braak & Verdonschot, 1995). All density and physicochemical parameters were log10(*X *+* *1) transformed to meet assumptions of multivariate normality and to moderate the influence of extreme data (Borcard, Gillet, & Legendre, 2011). CANOCO 4.5 was used to perform all the ordinations (ter Braak & Verdonschot, 1995).

## 3 RESULTS

### 3.1 Temporal and spatial change in species number

Macrozoobenthos samples from Poyang Lake Basin were classified into 81 species, 28 families, 9 classes, and 3 phyla. 64.2% of Mollusca, 23.5% of Arthropoda, and 12.3% of Annelida in the total number of macrozoobenthos species were found in Poyang Lake Basin (Table S1). The dominant species were *Chironmus* sp., *Nephtys oligobranchia*,* Bellamya purificata*,* Rivularia auriculata*,* Limnoperna lacustris,* and *Corbicula fluminea*. The sampling completeness was relatively high, with the Chao I measures estimator indicating more than 95% completeness at each sampling section and in each season. The final slopes of the observed and estimated species accumulation curves for macrozoobenthos at each section and each season were close to be asymptotic (Figure S3).

Significant differences were detected among the number of macrozoobenthos species in Poyang Lake Basin during different seasons (ANOVA, *p < *0.05). The number of macrozoobenthos species in summer was the greatest (51), followed by autumn (41), and the number of macrozoobenthos species was the lowest in winter (23; Table 1). In addition, we also found significant differences in the number of species among each section (ANOVA, *p < *0.05). The number of macrozoobenthos species in the “five rivers” was the greatest (57), followed by the main lake area of Poyang Lake (48). The middle reach of the Yangtze River contained the lowest number of species (12; Table 1).

### 3.2 Temporal and spatial changes in density and biomass

**Figure 2:**
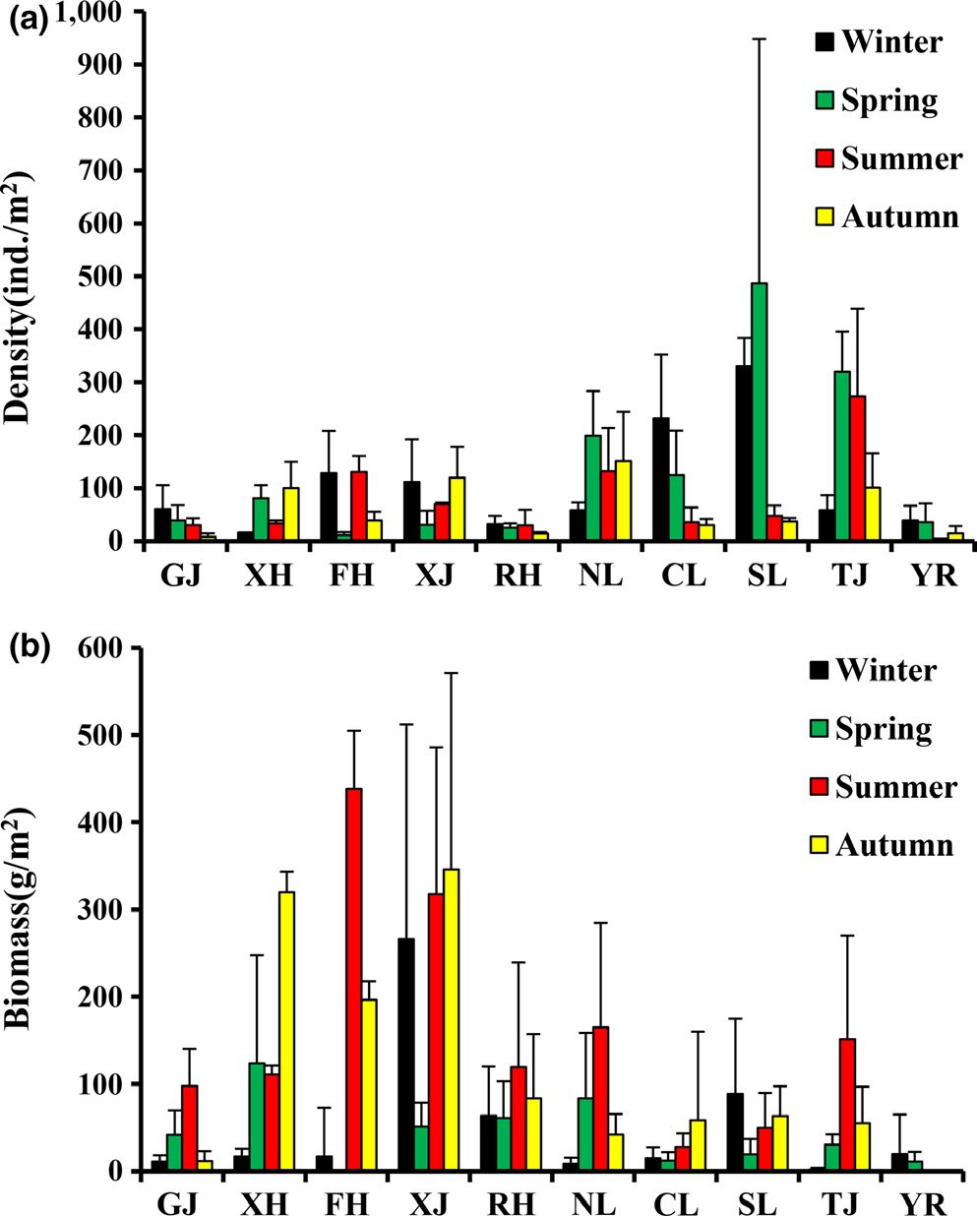
Temporal change in the density (a) and biomass (b) of macrozoobenthos in Poyang Lake Basin

**Figure 3:**
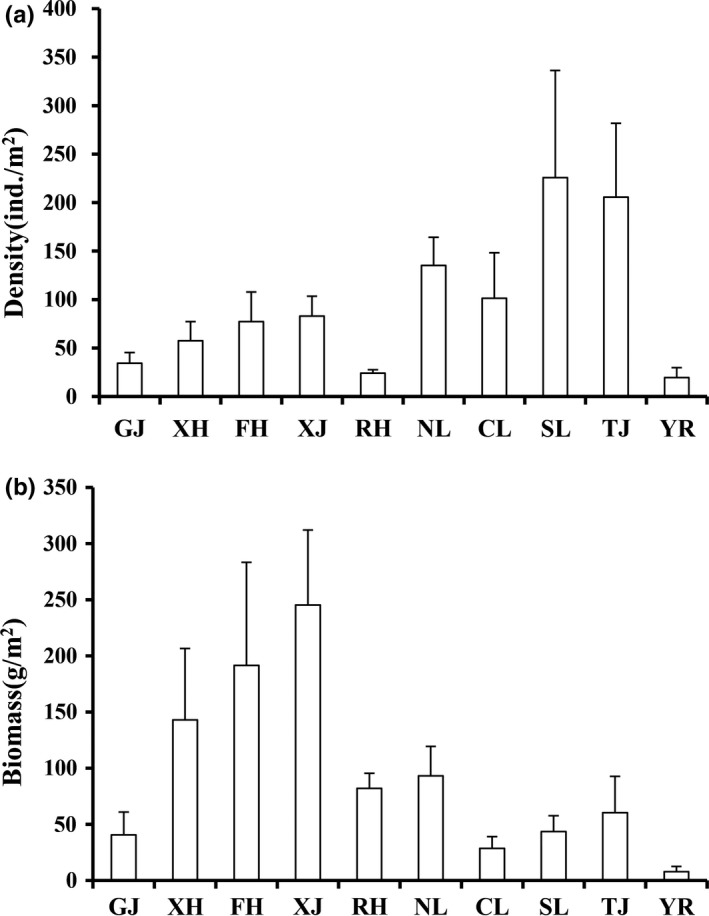
Spatial change in the density (a) and biomass (b) of macrozoobenthos in Poyang Lake Basin

**Figure 4:**
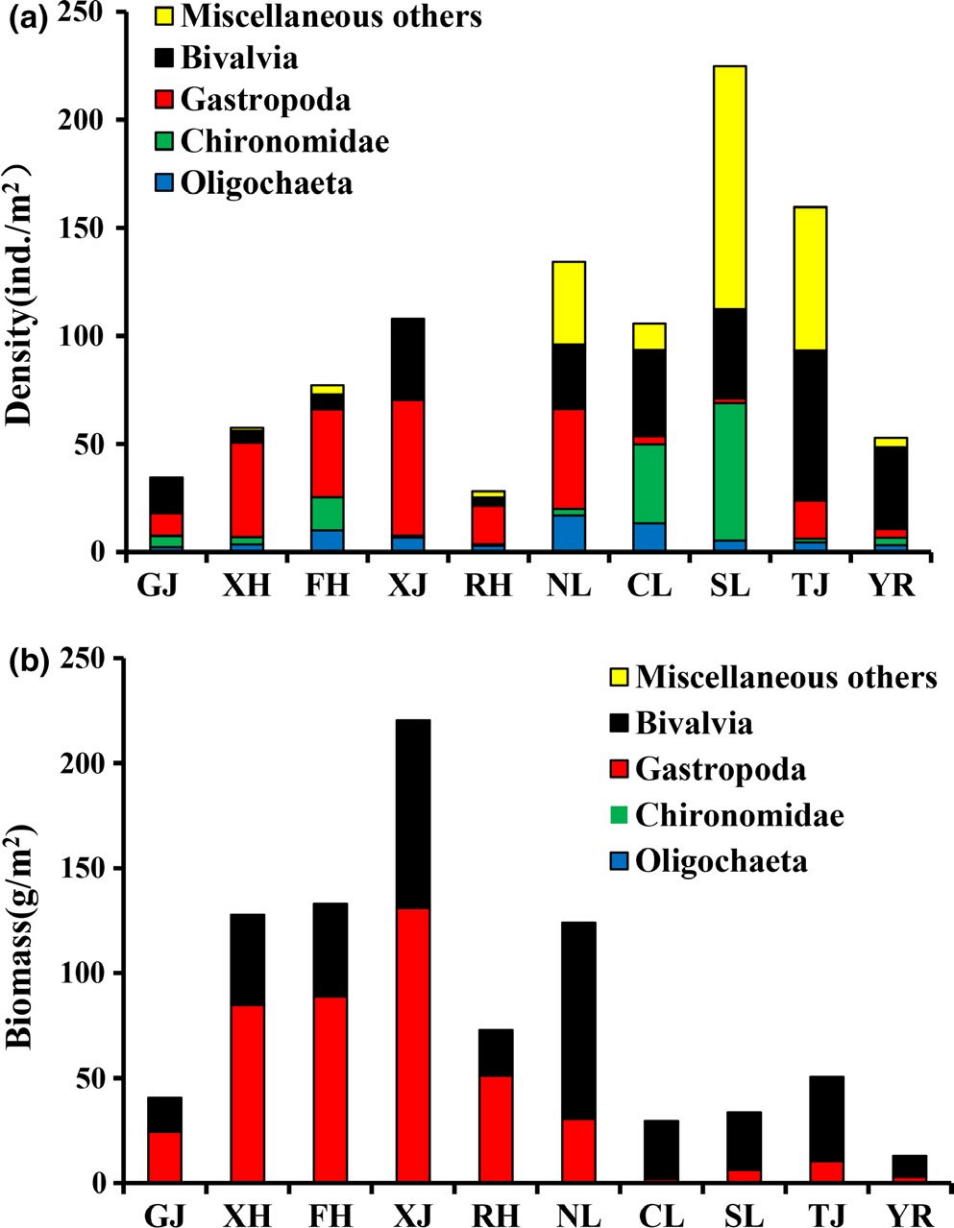
Composition of the density (a) and biomass (b) of different macrozoobenthos taxa in Poyang Lake Basin

The mean density and biomass of macrozoobenthos in Poyang Lake Basin were 100.6 ind./m^2^ and 65.8 g/m^2^, respectively. Significant differences were detected in the density and biomass of macrozoobenthos among the seasons (ANOVA, *p < *0.05). The density of macrozoobenthos in spring was the greatest (142.2 ind./m^2^), followed by winter (111.0 ind./m^2^); the density of macrozoobenthos in autumn was the lowest (61.1 ind./m^2^; Figure 2a). The biomass of macrozoobenthos in autumn was the greatest (174.7 g/m^2^), followed by summer (112.3 g/m^2^), and the macrozoobenthos biomass was the lowest in the spring (61.1 ind./m^2^; Figure 2b). In addition, we also found significant differences in the biomass among different sections (ANOVA, *p < *0.05). The density of macrozoobenthos in the connected‐river channel was the greatest (205.6 ind./m^2^), followed by the southern area of Poyang Lake (168.0 ind./m^2^), and the biomass was the lowest in the middle reach of the Yangtze River (23.00 ind./m^2^; Figure 3a). The macrozoobenthos biomass in the Xinjiang River was the greatest (245.3 g/m^2^), followed by the Fuhe River (191.5 g/m^2^), and the macrozoobenthos biomass was the lowest in the middle reach of the Yangtze River (7.79 g/m^2^; Figure 3b). The density and biomass of Gastropoda and Bivalvia in “five rivers” were greater than other sections. The density of Chironomidae, Oligochaeta, and Miscellaneous species in the main lake area of Poyang Lake was greater than that in other sections, but their biomass in these sections was lower than that in other sections (Figure 4).

### 3.3 Temporal and spatial changes in diversity

**Figure 5:**
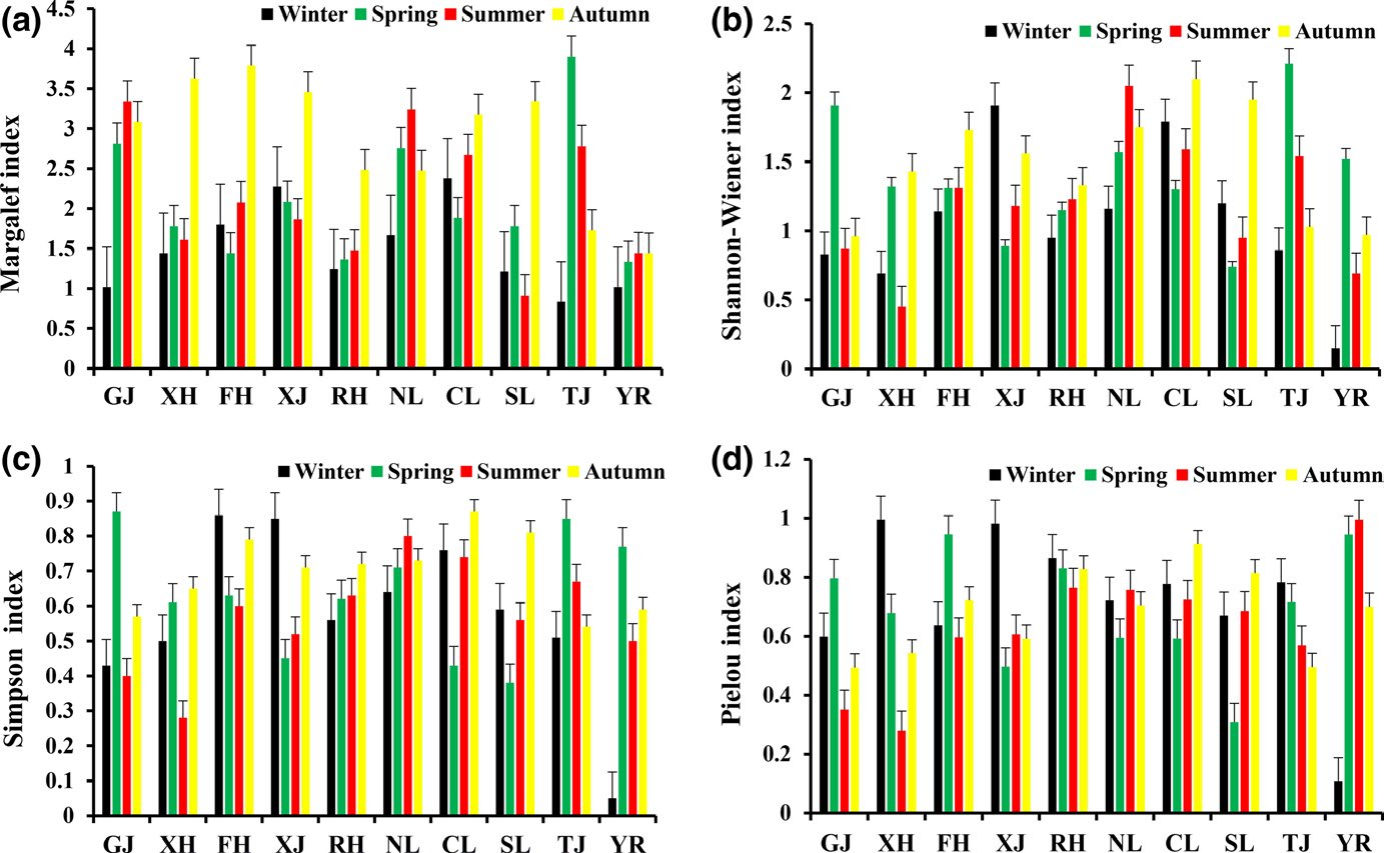
Temporal change in the diversity of macrozoobenthos in Poyang Lake Basin

**Figure 6:**
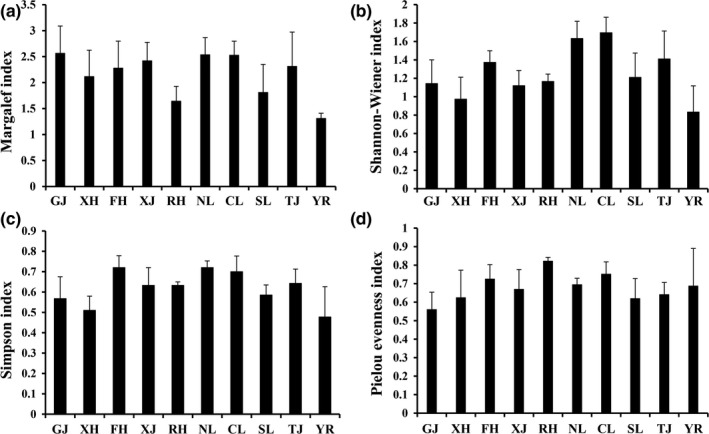
Spatial change in the diversity of macrozoobenthos in Poyang Lake Basin

Significant differences were detected in the diversity of macrozoobenthos among different seasons (ANOVA, *p < *0.05). The macrozoobenthos abundance and diversity in summer and autumn were greater than those in other seasons (Figure 5). We also found significant differences between the sections (ANOVA, *p < *0.05). The abundance and diversity in the main lake area of Poyang Lake and the “five rivers” were greater than those in other sections (Figure 6).

**Figure 7:**
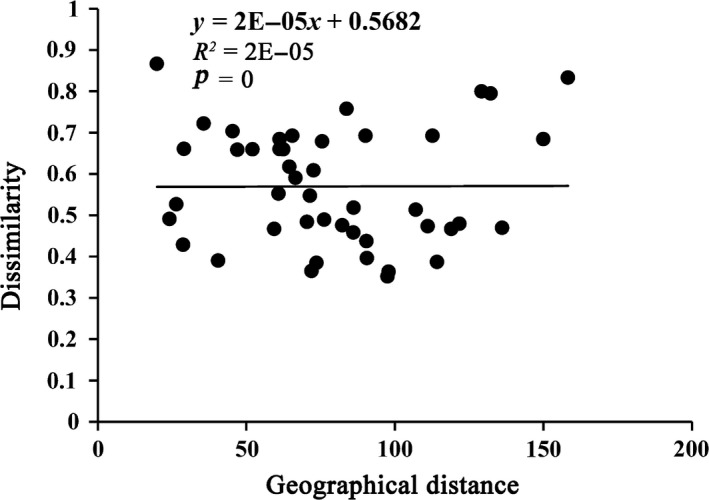
Effects of geographical distance on pairwise compositional dissimilarity components of macrozoobenthos obtained from BAS frameworks in Poyang Lake Basin

The macrozoobenthos composition dissimilarity was high with a mean value of 0.57 (Table 2). The spatial turnover component (0.44) was greater than the nestedness component (0.13). YR and SL exhibited high composition dissimilarities (0.66 and 0.68), a high spatial turnover (0.56) was found in SL, and the nestedness component (0.23) was exhibited in TJ (Table 2). In addition, the macrozoobenthos composition dissimilarity in winter (0.39) was higher than that in other seasons. In addition to spring, the spatial turnover component in other seasons was greater than the nestedness component. We found a significant effect of geographical distance on the overall beta diversity in Poyang Lake Basin (*p < *0.05; Figure 7).

**Table 2:** Temporal and spatial change in the beta diversity of macrozoobenthos in Poyang Lake Basin

	*β*_sor_	*β*_sim_	*β*_sne_
Spatial change			
GJ	0.50 ± 0.11	0.39 ± 0.11	0.11 ± 0.06
XH	0.55 ± 0.13	0.45 ± 0.17	0.10 ± 0.08
FH	0.53 ± 0.17	0.40 ± 0.15	0.13 ± 0.07
XJ	0.56 ± 0.18	0.47 ± 0.19	0.09 ± 0.05
RH	0.58 ± 0.16	0.45 ± 0.20	0.12 ± 0.08
NL	0.50 ± 0.10	0.35 ± 0.08	0.15 ± 0.09
CL	0.59 ± 0.09	0.48 ± 0.17	0.11 ± 0.09
SL	0.66 ± 0.12	0.56 ± 0.15	0.10 ± 0.08
TJ	0.56 ± 0.11	0.33 ± 0.09	0.23 ± 0.12
YR	0.68 ± 0.14	0.48 ± 0.21	0.20 ± 0.13
Temporal change			
Winter	0.39 ± 0.03	0.29 ± 0.14	0.10 ± 0.09
Spring	0.30 ± 0.06	0.08 ± 0.06	0.22 ± 0.05
Summer	0.30 ± 0.07	0.20 ± 0.13	0.10 ± 0.07
Autumn	0.34 ± 0.08	0.22 ± 0.19	0.12 ± 0.10

### 3.4 Assemblage structure of macrozoobenthos

**Figure 8:**
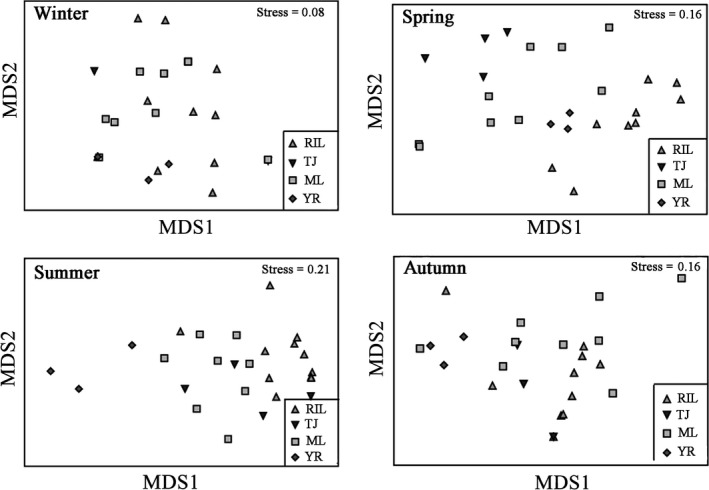
Metric multidimensional scaling (MDS) ordination of the macrozoobenthos community in Poyang Lake Basin. RIL: the lower of the “five rivers”; TJ: the connected‐river channel of Poyang Lake; ML: the main lake area of Poyang Lake; YR: the middle reach of the Yangtze River

MDS showed that the assemblage structure of macrozoobenthos in Poyang Lake Basin was similar in winter (Figure 8). The assemblage structure of macrozoobenthos in spring was divided into two areas, in which the first area included the connected‐river channel and the main lake area of Poyang Lake and the second area included the middle reach of the Yangtze River and the “five rivers” (Figure 8). The assemblage structure was divided into two areas in summer and autumn, in which the first area included the connected‐river channel, the main lake area of Poyang Lake, and the “five rivers” and the second area included the middle reach of the Yangtze River (Figure 8).

### 3.5 Correlation between macrozoobenthos assemblage structure and physicochemical parameters

**Figure 9:**
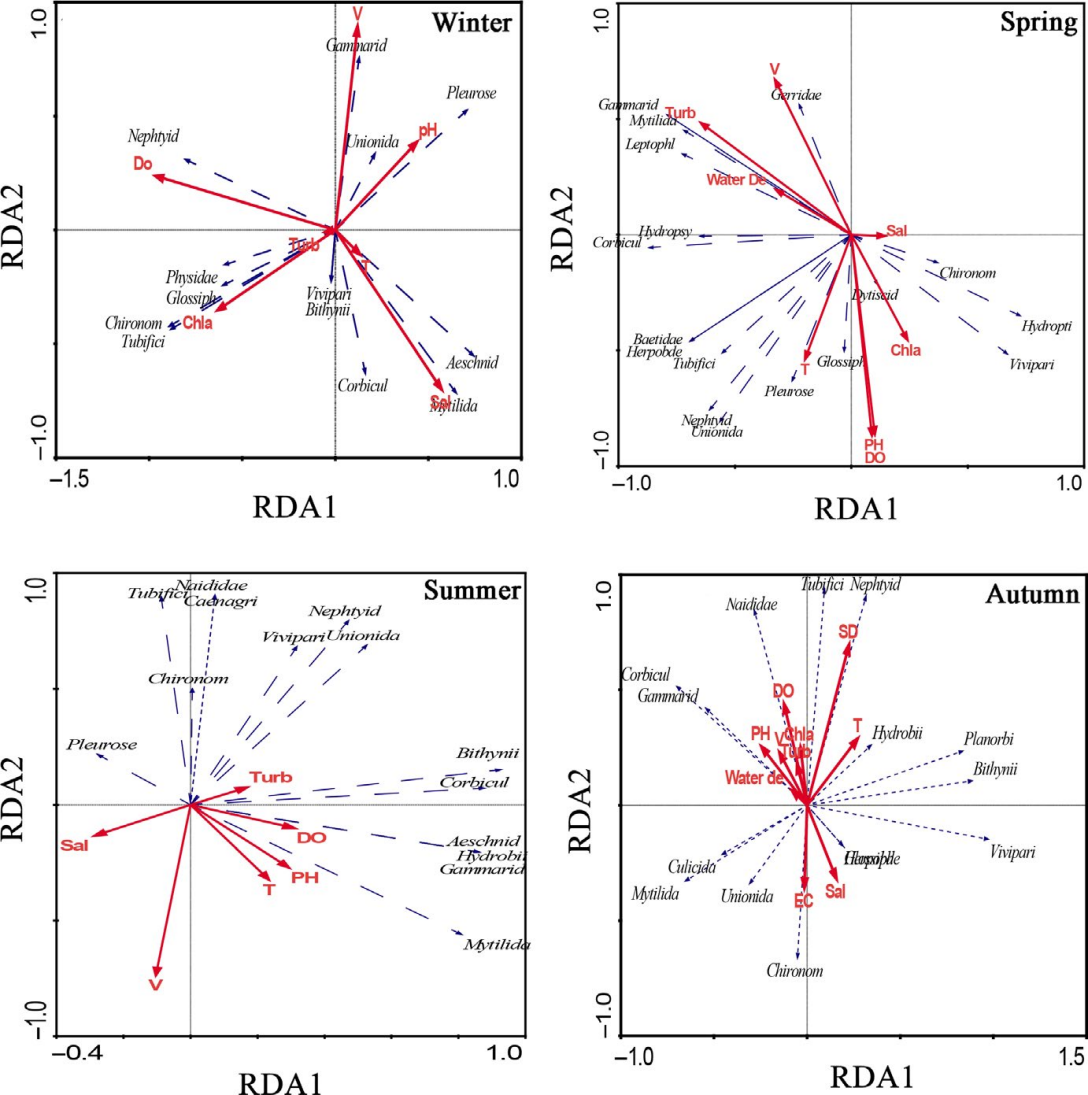
Ordination biplot of macrozoobenthos species assemblages and environmental variables obtained by RDA across sampling periods and sites. (T: temperature; TURB: turbidity; DO: dissolved oxygen; D: water depth; V: velocity; Sal: salinity; Chl‐a: chlorophyll‐a)

Significant differences were detected among the water depth, turbidity, chlorophyll‐a, and velocity in Poyang Lake Basin during different seasons (ANOVA, *p < *0.05; Table 3). No significant differences were detected in the temperature, dissolved oxygen, pH, and salinity during different seasons (ANOVA, *p* < 0.05). We also found significant differences in the water depth, temperature, chlorophyll‐a, and velocity between different seasons (ANOVA, *p < *0.05; Table 3). No significant differences were detected in the dissolved oxygen, turbidity, pH, and salinity between different seasons (ANOVA, *p* < 0.05). RDA showed that the phylum Mollusca was correlated with differences in the dissolved oxygen, pH, and salinity; Arthropoda was correlated with differences in the velocity, dissolved oxygen, and water depth; and Annelida was correlated with differences in the dissolved oxygen and chlorophyll‐a. Therefore, the water depth, dissolved oxygen, water velocity, and chlorophyll‐a significantly affected the distribution and assemblage structure of macrozoobenthos (Figure 9).

**Table 3:** Mean physicochemical parameters of water quality from 28 sampling sections in Poyang Lake Basin (mean ± *SE*)

Parameters	GJ	XH	FH	XJ	RH	NL	CL	SL	TJ	YR
	Mean ± *SD*	Mean ± *SD*	Mean ± *SD*	Mean ± *SD*	Mean ± *SD*	Mean ± *SD*	Mean ± *SD*	Mean ± *SD*	Mean ± *SD*	Mean ± *SD*
T(°)	19.9 ± 3.0	20.2 ± 3.1	19.5 ± 3.3	18.8 ± 3.3	18.3 ± 2.7	21.0 ± 2.7	18.9 ± 3.9	17.9 ± 3.0	20.0 ± 3.3	20.3 ± 2.9
pH	7.2 ± 0.6	7.0 ± 0.5	6.5 ± 1.3	6.8 ± 0.8	6.8 ± 0.9	6.7 ± 0.5	6.7 ± 0.6	6.8 ± 0.5	6.8 ± 0.5	6.6 ± 0.6
DO (mg/L)	8.9 ± 0.2	8.8 ± 0.5	9.8 ± 0.4	8.9 ± 0.8	8.6 ± 0.7	9.4 ± 0.3	9.1 ± 0.7	9.1 ± 0.6	9.6 ± 0.4	9.0 ± 0.2
TURB (NTU^+^)	18.3 ± 2.9	42.2 ± 2.2	24.5 ± 13.6	13.9 ± 1.2	42.8 ± 9.6	47.5 ± 17.5	53.6 ± 19.4	33.2 ± 12.1	66.7 ± 20.6	72.0 ± 26.5
Sal (mg/L)	0.05 ± 0.01	0.04 ± 0.01	0.03 ± 0.01	0.04 ± 0.01	0.05 ± 0.02	0.05 ± 0.01	0.04 ± 0.01	0.04 ± 0.01	0.06 ± 0.01	0.11 ± 0.02
V (m/s)	0.20 ± 0.04	0.10 ± 0.06	0.10 ± 0.03	0.10 ± 0.05	0.10 ± 0.04	0.26 ± 0.09	0.28 ± 0.08	0.21 ± 0.13	0.30 ± 0.14	0.30 ± 0.08
Chl‐a (μg/L)	18.0 ± 4.8	29.0 ± 7.3	37.2 ± 14.0	11.2 ± 2.7	19.8 ± 3.2	15.5 ± 6.7	13.8 ± 0.5	18.2 ± 5.3	14.1 ± 2.7	7.4 ± 1.9
WD (m)	–	–	–	–	–	6.1 ± 1.4	5.9 ± 1.6	4.7 ± 0.4	12.6 ± 1.0	16.4 ± 0.3

Abbreviations: T: temperature; TURB: turbidity; DO: dissolved oxygen; D: water depth; V: velocity; Sal: salinity; Chl‐a: chlorophyll‐a

## 4 DISCUSSION

### 4.1 Changes in the diversity of macrozoobenthos in Poyang Lake Basin

**Figure 10:**
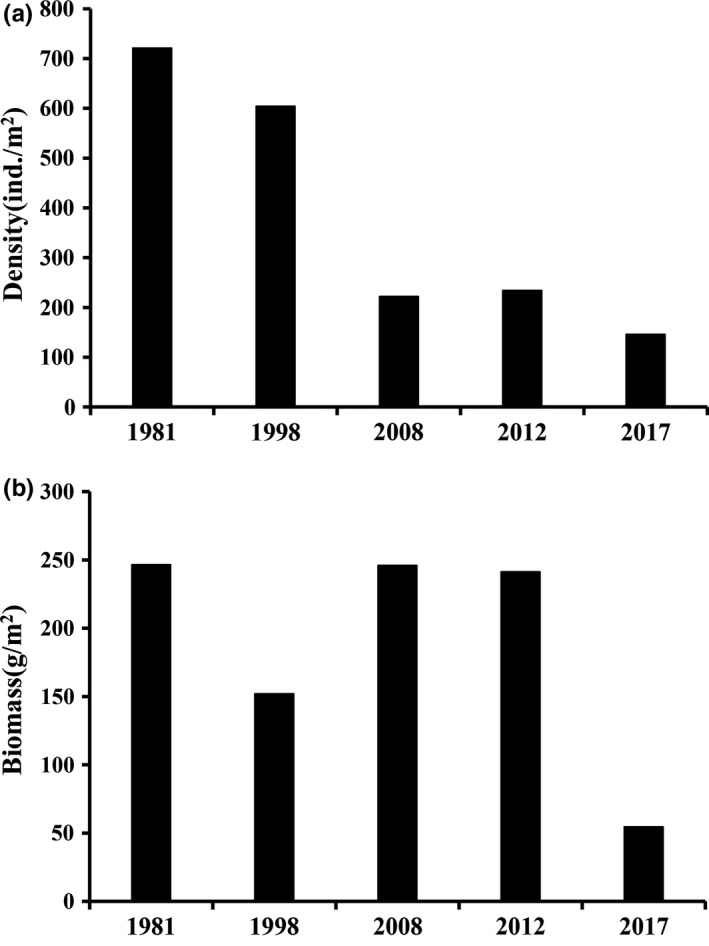
Change in the density (a) and biomass (b) of macrozoobenthos in Poyang Lake Basin

The number of macrozoobenthos species in Poyang Lake Basin in this study was lower than that indicated by historical data (Wang et al., 1999; Xie et al., 1995). There were 108 species of Mollusca in Poyang Lake, of which more than 50% were Chinese endemic species (Lin, 1962; Tchang & Li, 1965; Xiong et al., 2012). However, due to sand mining, overfishing and water pollution, many Mollusca populations have seriously declined in number (Shu, Wang, Pan, Liu, & Wang, 2009; Zhang et al., 2013). In addition, the density and biomass of macrozoobenthos in this study declined when compared to the historical data. In particular, their density and biomass declined by 80% and 70%, respectively (Figure 10; Xie et al., 1995; Wang et al., 1999; Ouyang et al., 2009; Cai et al., 2014), which may be attributed to sand mining. Poyang Lake contributed 2.4 × 10^8^ m^3^ sand, which accounted for 9.1% of the total consumed sand in China (Meng et al., 2018), leading to an annual extraction of sand that is 20 times greater than the natural sediment deposition (mean 1.03 × 10^7^ m^3^ per year; Leeuw et al., 2010). Sand mining has changed the physicochemical properties of water and aquatic organism habitats, which has greatly affected the macrozoobenthos community structure (Johnson, Jin, Carreiro, & Jack, 2012; Lewis, Weber, Stanley, & Moore, 2001; Narin & Michel, 2009). In addition, the dominant taxa of bivalves have shifted from large unionids to the small *Corbicula fluminea* (Cai et al., 2014; Ouyang et al., 2009; Shu et al., 2009).

### 4.2 Spatial heterogeneity of diversity of macrozoobenthos

The macrozoobenthos community structure is determined by the spatial difference between complexity of habitats (Shostell & Williams, 2007; Tews et al., 2004). In this study, the macrozoobenthos density, biomass, and diversity in different regions of Poyang Lake Basin showed spatial heterogeneity (*p < *0.05). Many studies have shown that the diversity of shellfish in rivers is significantly higher than that in lakes (Vaughn, 2012; Xiong et al., 2012). We also found that the diversity of shellfish in the lower reach of the “five rivers” was higher than that in other regions, as shellfish were more likely to live in a habitat with a low slope, sandy transitional zone, and a small amount of vegetation cover (Liu, 2013; Zhang et al., 2013). The nutrient level and sediment sizes directly or indirectly affected the macrozoobenthos community structure (Beisel, Usseglio‐Polatera, & Moreteau, 2000; Gao & Yin, 2010). Some studies have shown that finer substrates and simple microhabitats in the lower reaches of the river could lead directly to a decline in the abundance of Ephemeroptera, Plecoptera, and Trichoptera (Beisel et al., 2000; Flecker & Feifarek, 1994; Newell, Hitchcock, & Seiderer, 1999). In this study, the density and biomass of the aquatic insects, Chironomus and Oligoshagus, in the main lake area of Poyang Lake were higher than those in other areas.

Changes in water level have significantly affected the macrozoobenthos community structure in rivers and lakes (Baudo, Ochhipinti, & Nocentini, 2001; Beisel et al., 2000; Petridis & Sinis, 1993). In this study, the macrozoobenthos community structure showed spatial heterogeneity based on MDS analysis. As the water level rises, the dissolved oxygen and water temperature decrease, and organic salt deposition causes eutrophication in the flood season in Poyang Lake, which has an effect on the density and biomass of macrozoobenthos, especially mussels and snails (Haag, 2012; Xu, 2013). In summer, bodies of water exchange frequently, and the area of the Poyang Lake Basin increases, which increases habitat heterogeneity, and the dominant species of macrozoobenthos in different habitats are significantly different (McCarthy, Bailey, & Estabroaks, 1998). For example, the organic matter in silt is richer and more diverse than that in other substrates, which makes the species diversity of macrozoobenthos more abundant and diverse in areas containing silt (Beauger, Lair, Reyes‐Marchant, & Peiry, 2006; Haag, 2012; Vaughn, 2012). In this study, the dominant taxa of macrozoobenthos in the main lake area of Poyang Lake were Chironomidae, Oligochaeta, and Miscellaneous others; the dominant taxa in the “five river” were Gastropoda and Bivalvia; and the dominant species in the middle reach of the Yangtze River was *Limnoperna lacustris*.

### 4.3 Key environmental factors determining the macrozoobenthos community structure

Aquatic habitats and their physicochemical parameters are important factors affecting the macrozoobenthos community structure. Some studies have shown that environmental factors such as water temperature, DO, turbidity, and flow velocity affect the macrozoobenthos community structure (Allan & Castillo, 2007; Wang et al., 2012). For example, the dissolved oxygen and electrical conductivity are the main factors affecting the distribution of aquatic insects and oligochaetes (Chen, Gao, Liu, Sun, & Kang, 2013; Wang et al., 2012; Zhang, Xu, Ma, Zhang, & Wang, 2007). The flow velocity also affects the distribution of macrozoobenthos, such as Oligochaetes and Chironomidae, which are more abundant in slow flowing water (Allan & Castillo, 2007). Chlorophyll‐a mainly affects the distribution of aquatic insects and oligochaetes, such as *Limnodrilus hoffmeisteri* and Chironomus, which are more abundant in eutrophic areas (Gong, Xie, & Tang, 2001). In this study, the water depth, dissolved oxygen, flow velocity, and chlorophyll‐a were significantly correlated with the distributions and assemblage structure of macrozoobenthos based on RDA.

### 4.4 Effect of human activity on the diversity of macrozoobenthos

The aquatic ecosystem of Poyang Lake Basin is undergoing habitat degradation, which leads to a decline in biodiversity (Huang, Wu, & Li, 2013; Jin et al., 2012; Xiong et al., 2012). The degradation process is driven by human intervention and natural factor in the basin (Huang et al., 2013; Jin et al., 2012). These human activities mainly include sand mining, dam construction, water pollution, eutrophication, overfishing, and climate change (Cardinale et al., 2012; Jin et al., 2012).

With the acceleration of urbanization and the increase of the population in Poyang Lake Basin, the continuous input of industrial wastewater and domestic sewage, and the increasing concentrations of nutrients and heavy metals have resulted in water quality deterioration and eutrophication and indirectly affected macrozoobenthos community structure (Hu, Zhou, Wang, & Wei, 2010; Wan & Jiang, 2005). The direct impact of sand mining includes the removal of sandbed resources (Hitchcock & Bell, 2004), changes to the sandbed topography and sediment composition (Cooper et al., 2007), water pollution (Hancock, 2002), a decline in the surface area of hydrophytes (Erftemeijer & Lewis, 2006), and a reduction in the abundance of macrozoobenthos (Boyd, Limpenny, Rees, & Cooper, 2005). Some studies have shown that sand mining can cause losses of 30%–70% in terms of species richness and 40%–95% in terms of abundance and biomass (Desprez, 2000). Sand mining has caused habitat fragmentation and deterioration in Poyang Lake Basin, which has endangered many Mollusca species, and the dominant species are gradually becoming miniaturized (Shu et al., 2009; Xiong et al., 2012; Zhang et al., 2013). Moreover, the dams in the upstream reaches of “five rivers” resulted in significantly changing hydrological conditions, thereby influencing the macrozoobenthos community structure (Liu, Hu, Ao, Wu, & Ouyang, 2017).

### 4.5 Conservation and management implications

Given the above key factors driving the degradation of the ecosystem and the decline in biodiversity in Poyang Lake Basin, we provide some conservation measures: (a) The regulation and management of sand mining, such as limiting sediment screening in Poyang Lake Basin, would reduce water turbidity and changes in the sediment composition of the lake and riverbed, and setting an appropriate depth for sanding mining could help avoid intense changes in the composition of the faunal community. At the same time, ongoing environmental monitoring of sand mining projects can help provide reassurance that the impacts of sand mining are in line with predictions made during the Environmental Impact Assessment (EIA). (b) The natural hydrological rhythm (dam release discharge) should be maintained to keep the balance and uniformity in time and space during each year to regulate the water level for water conservation projects in Poyang Lake Basin. This is conducive to the survival and reproduction of macrozoobenthos. (c) The fixed point treatment of domestic refuse and industrial waste in the river basin should be conducted as far as possible before being discharged into the river.

## CONFLICT OF INTEREST

The authors declare that there are no conflicts of interest.

## AUTHOR CONTRIBUTIONS

LK, LXJ, OYS, and WXP conceived the study. All authors contributed to the study design and data collection. LK and LXJ analyzed the data. LK, LXJ, OYS, and WXP led the writing of the manuscript.

## Data Availability

The data used in this manuscript were obtained from field investigations and laboratory experiments (taxon composition). The author has attached the taxon information in supplemental files. Please see Table S1.
