# Supplementary figures and images for: Temporal and spatial changes in macrozoobenthos diversity in Poyang Lake Basin, China
Source: Ecol Evol. 2019 Apr 26;9(11):6353–65. doi: 10.1002/ece3.5207 (PMC6580267; doi:10.1002/ece3.5207)

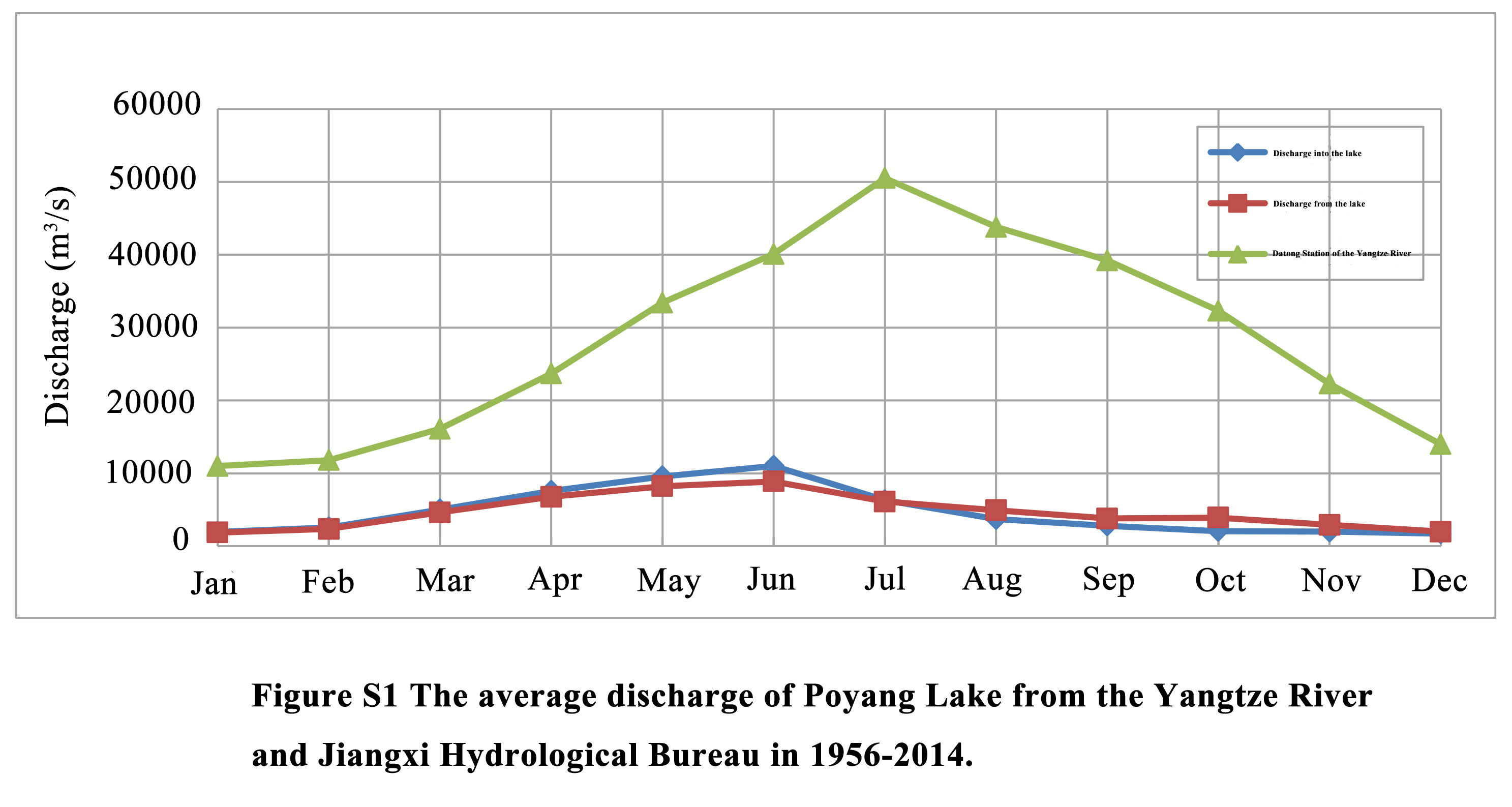

Supplement: Supplementary file 1 [file ECE3-9-6353-s001.tif]

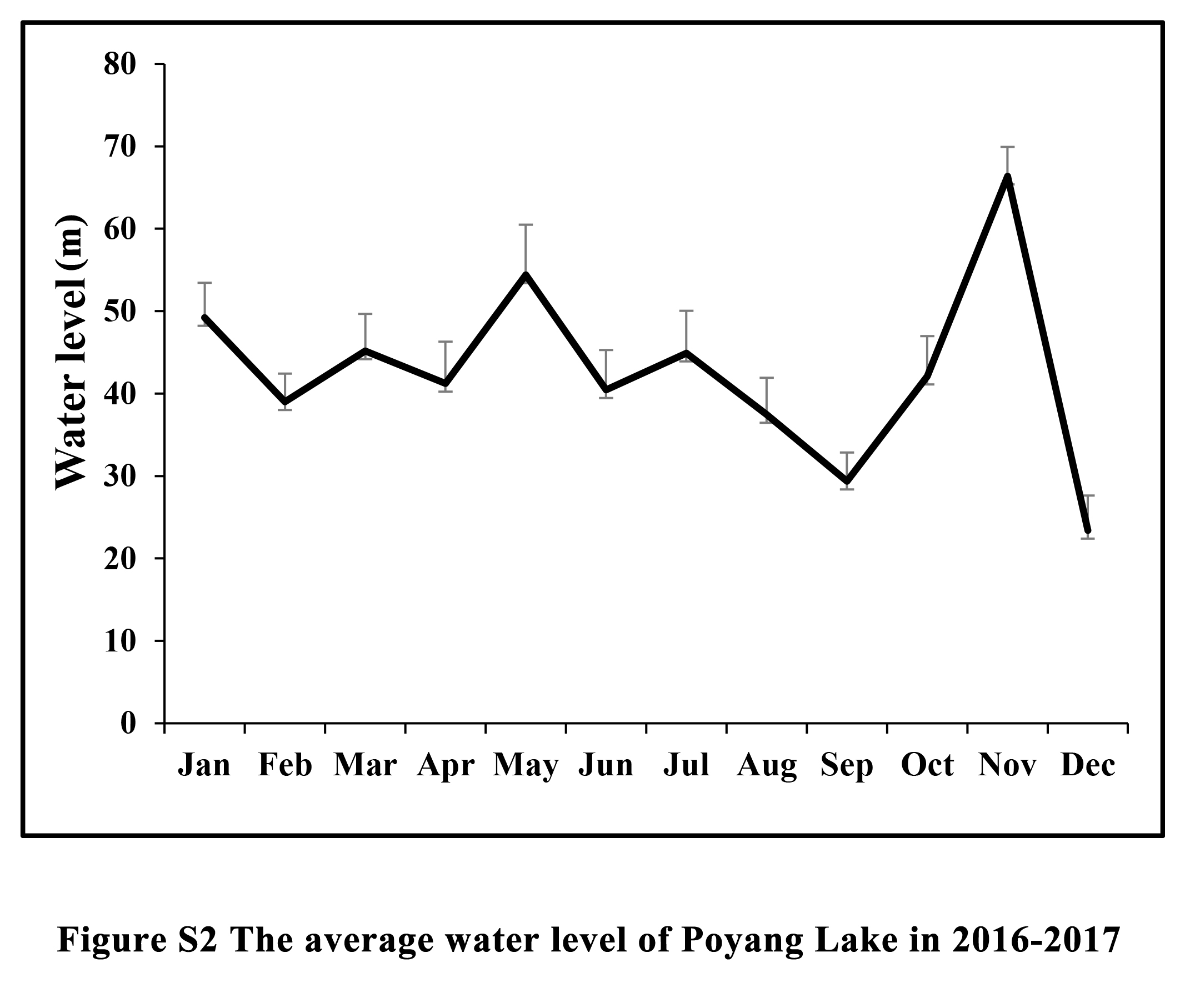

Supplement: Supplementary file 2 [file ECE3-9-6353-s002.tif]

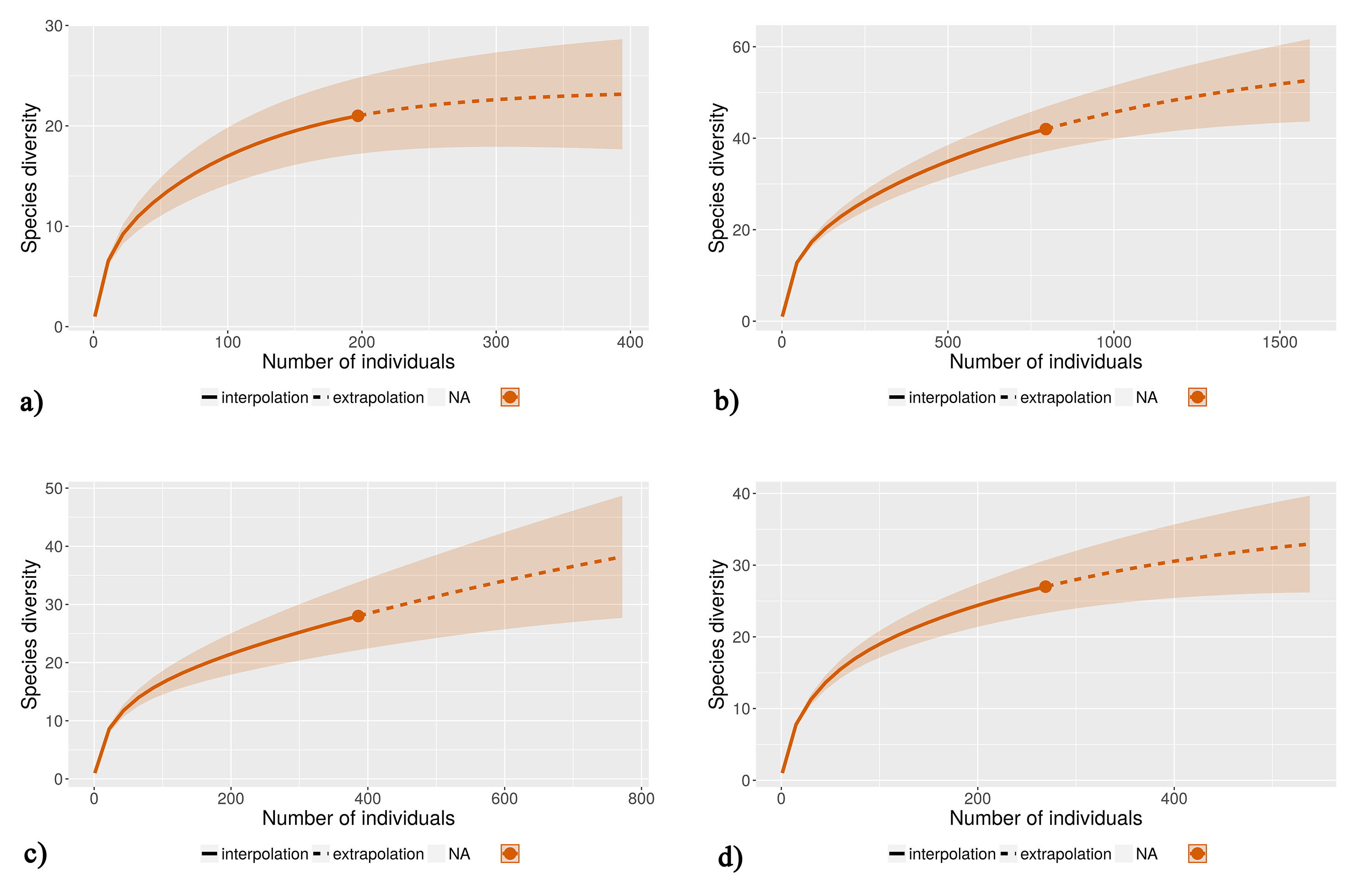

Supplement: Supplementary file 3 [file ECE3-9-6353-s003.tif]
