# Supplementary material for: Temporal and spatial changes in macrozoobenthos diversity in Poyang Lake Basin, China
Source: Ecol Evol. 2019 Apr 26;9(11):6353–65. doi: 10.1002/ece3.5207 (PMC6580267; doi:10.1002/ece3.5207)
Supplement: Supplementary file 4 [file ECE3-9-6353-s004.docx]

**Table S1**

Distribution and composition of species macrozoobenthos in Poyang Lake Basin.

| Taxa | GJ | XH | FH | XJ | RH | NL | CL | SL | T J | Y R |
| --- | --- | --- | --- | --- | --- | --- | --- | --- | --- | --- |
| **Annelida** |  |  |  |  |  |  |  |  |  |  |
| **Polychaota** |  |  |  |  |  |  |  |  |  |  |
| *Nephtys oligobranchia* |  |  | + |  |  | + | + | + | + |  |
| **Oligochaeta** |  |  |  |  |  |  |  |  |  |  |
| **Naididae** |  |  |  |  |  |  |  |  |  |  |
| *Nais* sp. |  |  |  |  |  | + |  |  | + |  |
| *Slavina* sp. |  |  |  |  |  |  |  |  | + |  |
| **Tubificidae** |  |  |  |  |  |  |  |  |  |  |
| *Branchiura sowerbyi* |  | + |  | + |  | + | + |  | + |  |
| *Tubifex sinicus* |  |  |  |  |  | + | + |  | + | + |
| *Limnodrilus hoffmeisteri* | + | + | + | + | + | + | + | + | + | + |
| *Limnodrilus claparedianus* |  |  |  |  |  | + |  |  |  |  |
| *Aulodrilus prothecatus* |  |  |  |  |  |  |  |  | + |  |
| **Hirudinea** |  |  |  |  |  |  |  |  |  |  |
| **Glossiphonidae** |  |  |  |  |  |  |  |  |  |  |
| *Glossiphonia* sp. |  | + |  |  | + | + | + |  |  |  |
| **Herpobdellidae** |  |  |  |  |  |  |  |  |  |  |
| *Herpobdella* sp. |  | + |  |  |  |  |  |  | + |  |
| **Mollusca** |  |  |  |  |  |  |  |  |  |  |
| **Gastropoda** |  |  |  |  |  |  |  |  |  |  |
| **Viviparidae** |  |  |  |  |  |  |  |  |  |  |
| *Bellamya aeruginosa* | + | + | + | + | + | + |  |  |  |  |
| *Bellamya quadrata* |  |  |  | + |  |  |  |  |  |  |
| *Bellamya purificata* | + | + | + | + | + | + | + | + | + | + |
| *Cipangopaludina chinensis* |  |  |  |  |  |  |  |  |  |  |
| *Rivularia auriculata* | + | + | + | + |  |  |  |  |  |  |
| *Rivularia ovum* | + |  | + | + | + |  |  | + |  |  |
| **Hydrobiidae** |  |  |  |  |  |  |  |  |  |  |
| *Oncomelania hupensis hupensis* |  |  |  | + |  | + | + |  | + | + |
| **Bithyniidae** |  |  |  |  |  |  |  |  |  |  |
| *Alocinma longicornis* |  | + | + |  |  | + |  | + | + |  |
| *Parafossarulus striatulus* | + |  |  | + |  |  |  |  | + |  |
| *Parafossarulus anomalospiralis* |  | + | + | + |  | + | + |  | + |  |
| *Parafossarulus sinensis* |  |  |  |  |  |  |  |  | + |  |
| **Stenothyridae** |  |  |  |  |  |  |  |  |  |  |
| *Stenothyra toucheana* |  |  |  |  |  |  |  |  | + |  |
| **Pleuroseridae** |  |  |  |  |  |  |  |  |  |  |
| *Semisulcospira cancellata* | + |  | + | + |  | + | + |  | + | + |
| *Semisulcospira libertina* |  |  |  |  |  |  |  |  |  |  |
| **Physidae** |  |  |  |  |  |  |  |  |  |  |
| *Physa acuta* |  |  |  |  |  |  | + |  |  |  |
| **Planorbidae** |  |  |  |  |  |  |  |  |  |  |
| *Gyraulus compressus* |  |  |  |  |  |  |  |  | + |  |
| **Lamellibranchia** |  |  |  |  |  |  |  |  |  |  |
| **Mytilidae** |  |  |  |  |  |  |  |  |  |  |
| *Limnoperna lacustris* | + |  |  |  |  | + | + | + | + | + |
| **Corbiculidae** |  |  |  |  |  |  |  |  |  |  |
| *Corbicula fluminea* | + | + | + |  | + | + | + | + | + | + |
| **Unionidae** |  |  |  |  |  |  |  |  |  |  |
| *Anodonta woodiana* | + | + | + | + | + | + | + |  | + |  |
| *Anodonta woodiana elliptica* |  |  | + |  |  |  |  |  |  |  |
| *Anodonta woodiana pacifica* |  |  | + |  |  |  |  |  | + |  |
| *Anodonta rivularia* |  | + | + | + |  | + |  |  | + |  |
| *Anodonta euscaphys* |  |  | + |  |  | + |  |  | + |  |
| *Anodonta globosula* |  |  |  |  |  | + |  |  |  |  |
| *Anodonta arcaeformi* | + | + | + | + | + | + |  |  | + |  |
| *Anodonta arcaeformis flavotincta* |  |  |  |  |  | + |  |  |  |  |
| *Lamprotula rochechouarti* | + |  |  |  |  |  |  |  | + |  |
| *Lamprotula caveata* | + | + | + | + | + | + |  |  | + |  |
| *Lamprotula leai* |  |  | + |  |  | + |  |  | + |  |
| *Lamprotula microsticta* |  | + |  |  |  |  |  |  |  |  |
| *Lamprotula*  *zonata* |  |  |  | + | + |  |  |  | + |  |
| *Lamprotula polysticta* |  |  |  | + |  |  |  |  | + |  |
| *Lamprotula tortuosa* |  |  |  |  |  |  |  |  | + |  |
| *Aculamprotula fibrosa* |  |  |  |  |  |  |  |  | + |  |
| *Cuneopsis heudei* | + |  | + | + | + |  |  |  |  |  |
| *Cuneopsis pisciculus* | + |  | + | + |  |  |  |  | + |  |
| *Cuneopsis capitata* |  |  | + |  |  |  |  |  | + |  |
| *Cuneopsis celtiformis* | + |  |  |  | + |  |  |  |  |  |
| *Lanceolaria grayanus* |  |  | + | + |  | + |  |  | + |  |
| *Lanceolaria eucylindrca* | + |  | + | + | + | + | + |  | + |  |
| *Acuticosta chinensis* | + | + | + | + |  | + |  |  |  |  |
| *Acuticosta ovata* |  |  | + |  |  |  |  |  |  |  |
| *Schistodesmus lampreyanus* |  |  | + | + |  |  |  |  |  |  |
| *Unio douglasiae* | + | + | + | + | + | + |  |  | + |  |
| *Hyriopsis cumingii* | + | + | + |  | + | + |  |  | + |  |
| *Arconaia lancelata* | + |  | + | + |  | + |  |  | + |  |
| *Cristaria plicata* |  | + |  | + | + |  |  |  |  |  |
| *Lepidodesma languilati* | + |  | + |  |  | + |  |  | + |  |
| *Solenaia carinata* |  |  | + |  |  |  |  |  |  |  |
| *Solenaia oleivora* |  |  | + | + |  |  |  |  |  |  |
| *Solenaia triangularis* |  |  |  |  |  |  |  |  |  |  |
| **Solecurtidae** |  |  |  |  |  |  |  |  |  |  |
| *Novaculina chinensis* |  |  |  |  |  |  |  |  | + |  |
| **Arthropoda** |  |  |  |  |  |  |  |  |  |  |
| **Insecta** |  |  |  |  |  |  |  |  |  |  |
| **Baetidae** |  |  |  |  |  |  |  |  |  |  |
| *Cloeon dipterum* |  |  |  |  |  |  |  |  | + |  |
| **Leptophlebiidae** |  |  |  |  |  |  |  |  |  |  |
| *Leptophlebia* sp. |  |  |  |  |  |  |  |  | + |  |
| **Caenagrionidae** |  |  |  |  |  |  |  |  |  |  |
| *Caenagrionidae* sp. |  |  |  |  |  | + |  |  |  |  |
| **Aeschnidae** |  |  |  |  |  |  |  |  |  |  |
| *Aeschna* sp. |  |  |  |  |  |  |  |  | + | + |
| **Gerridae** |  |  |  |  |  |  |  |  |  |  |
| *Gerris* sp. |  |  |  |  |  |  |  | + |  |  |
| **Dytiscidae** |  |  |  |  |  |  |  |  |  |  |
| *Agabus* sp. |  |  | + |  |  | + |  |  |  |  |
| *Cybister* sp. |  |  |  |  |  |  |  | + |  |  |
| **Hydroptilidae** |  |  |  |  |  |  |  |  |  |  |
| *Hydroptila* sp. |  |  | + |  |  |  |  |  |  |  |
| **Hydropsychidae** |  |  |  |  |  |  |  |  |  |  |
| *Hydropsyche* sp. |  |  |  |  |  |  | + |  |  |  |
| **Culicidae** |  |  |  |  |  |  |  |  |  |  |
| *Anopheles* sp. |  |  |  |  |  |  |  | + |  | + |
| **Chironomidae** |  |  |  |  |  |  |  |  |  |  |
| *Chironomus* sp. | + | + | + |  | + | + |  | + |  |  |
| *Tanytarsus* sp. |  |  | + |  |  |  | + | + |  |  |
| *Pelopia* sp. | + |  |  |  |  |  | + | + | + | + |
| *Endochironomus* sp. | + |  |  |  |  |  | + | + | + |  |
| *Clinotanypus* sp. | + |  |  |  |  |  |  | + | + |  |
| *Orthocladius* sp. |  | + |  |  |  |  |  | + |  | + |
| *Polypedilum* sp. |  |  |  |  |  | + |  | + |  |  |
| **Crustacea** |  |  |  |  |  |  |  |  |  |  |
| *Macrobrachium nipponensis* |  |  |  |  | + |  |  |  |  |  |
| *Gammarid* sp. |  |  |  |  |  | + | + | + | + | + |
